# Congenital Optic Disc Anomalies: Insights from Multimodal Imaging

**DOI:** 10.3390/jcm13051509

**Published:** 2024-03-06

**Authors:** Gilda Cennamo, Michele Rinaldi, Marina Concilio, Ciro Costagliola

**Affiliations:** 1 Department of Neurosciences, Reproductive Sciences and Dentistry, University of Naples “Federico II”, 80138 Napoli, Italy; ciro.costagliola@unina.it; 2 Department of Neurosciences, Reproductive and Odontostomatological Sciences, University of Naples “Federico II”, 80138 Napoli, Italy; 3 Department of Medicine and Health Sciences “V. Tiberio”, University of Molise, 86100 Campobasso, Italy; conciliomarina@gmail.com

**Keywords:** optic disc coloboma, optic disc pit, morning glory syndrome, optical coherence tomography, optical coherence tomography angiography

## Abstract

In this comprehensive review, we delve into the significance of multimodal imaging in diagnosing and managing complications of congenital optic disc anomalies. While the fundus examination is the gold standard tool in the diagnosis of these pathologies, spectral domain (SD) optical coherence tomography (OCT) and optical coherence tomography angiography (OCTA) could shed light on the pathogenesis and treatment. Moreover, this review seeks to offer a comprehensive insight into the multimodal approach of these rare congenital pathologies. In conclusion, congenital anomalies of the optic nerve represent a major challenge for ophthalmologists. Further research could be useful to clarify the pathophysiology of these diseases and define a correct and more specific treatment approach.

## 1. Introduction

Congenital optic disc anomalies or cavitary anomalies are a rare but important cause of visual impairment, with a visual prognosis ranging from asymptomatic to blindness [1]. This spectrum of pathologies includes optic disc coloboma (ODC), optic disc pit (ODP), and morning glory syndrome (MGS). The clinical presentation is usually unilateral, isolated, or associated with both ocular abnormalities and as part of systemic disorders [2].

**Figure 1:**
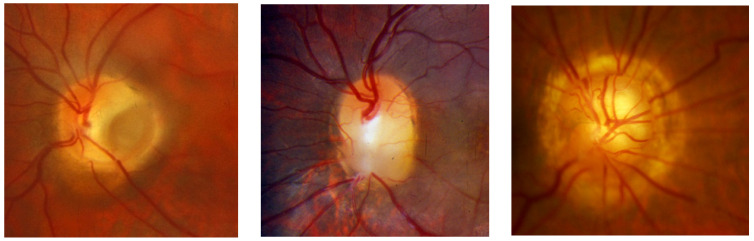
Colour fundus imaging of optic disc pit (ODP), optic disc coloboma (ODC), and morning glory syndrome (MGS) (from the left to right line).

Ophthalmologic examination with fundoscopy is usually sufficient to detect congenital optic nerve anomalies (Figure 1). However, these abnormalities can vary in disease severity and overlap clinically. Complementary imaging of the optic disc may increase the accuracy of diagnosis and potentially shed light on the pathophysiology of these disorders, which is still unclear [3]. Therefore, multimodal imaging with non-invasive exams may help to define the diagnosis, identify potential complications, and avoid unnecessary additional tests. Freund and colleagues reported a definition of multimodal imaging applied to the ophthalmic field, as follows: “the use of more than one technological system to acquire images, concurrently or at a short period of time, that complement one another for the purpose of diagnosis, prognostication, management, and monitoring of disease” [4]. Today, most reports classify multimodal imaging as the combination of en face fundus imaging, en face dye-based angiography, cross-sectional and/or en face optical coherence tomography (OCT), and possibly autofluorescence when relevant to evaluate a specific disease.

The aim of this review is to summarise the current literature on the pathophysiology, clinical manifestations, multimodal imaging for diagnosis, and possible treatments of complications associated with congenital optic disc anomalies.

## 2. Materials and Methods

From 20 November to 3 December 2023, a literature search was conducted by two authors (G.C, M.C.) across several research databases, including PubMed, Scopus, and Google Scholar. Keywords used to retrieve relevant documents included “anomalies of the optic disc”, “anomalies of nerve cavity”, “congenital anomalies of the optic disc”, “congenital anomalies of the optic nerve”, “optic nerve coloboma”, “optic disc coloboma”, “morning glory syndrome”, “morning glory disc anomaly”, “optic disc pit”. We only considered articles that responded to the above keywords and were published in the last 15 years. Exclusion criteria were non-English language articles, lack of access to full texts, and lack of access to full abstracts. We included case reports if there were no other research studies in the literature on the same specific topic.

## 3. Optic Disc Coloboma

ODC is the second most common congenital malformation, with a reported a prevalence of 8.9/100,000, which is not influenced by gender or race [5]. It is characterized by a typical aspect of bowl-shaped excavation with sharp borders, usually localized inferonasal, with a normal vasculature, and equally affected unilaterally or bilaterally [6]. When it occurs bilaterally, it can cause severe visual impairment in children with nystagmus, while when it occurs unilaterally or in asymmetric cases, visual function may not be significantly affected. Visual acuity is influenced by possible involvement of the fovea if retinoschisis or retinal detachment is present. It occurs sporadically but can be inherited in an autosomal dominant manner [6]. It can be isolated or in association with other ocular abnormalities, such as iris and/or retinal coloboma and aniridia in patients with the PAX6 mutation [7]. Several associations with genetic disorders have been described: PAX2 mutation-related papillorenal syndrome [8,9], CHARGE syndrome, and Aicardi syndrome [6,10].

Regarding the pathophysiology, it results from a defective closure of the proximal part of the embryonal fissure during the 6–7 weeks of gestation [1]. There are several animal models, which have been used to explore the genetic basis of ODC development. Beyond the role of PAX2 (which is one of the major players in optic nerve head development and optic fissure closure in the ventral retina), Yan and colleagues investigated the role of BMPR1B-mediated signalling in optic nerve development in a mouse model. They proved that a Bmpr1b mutation alters the expression pattern of PAX2 during embryonic development, leading to a delay in the closure of the optic fissure and then, to coloboma of the optic disc [11].

### 3.1. Multimodal Imaging

There are only a few studies on OCT in ODC. Structurally, SD-OCT shows a retinochoroidal–scleral excavation in the area of ODC and the presence of sclera directly under the retina, when a choroidal coloboma is present. If the ODC is complicated with retinoschisis, this will be visible above the excavation. Moreover, swept source (SS) OCT can detect the presence of herniated retinal tissue into the colobomatous area and the subarachnoid space immediately behind the highly reflective tissue lining, which may be the pia mater [12]. On optical coherence tomography angiography (OCTA), Cennamo and colleagues demonstrated the absence of a radial peripapillary microvascular network in ODC as a result of imperfect closure of the embryonic fessure [3].

Fluorescein angiography is useful in ODC complicated by choroidal neovascularization as it shows the presence of late hyperfluorescence at the margin of the optic nerve [13].

### 3.2. Complications

Less frequently than the other forms of optic disc anomalies, ODC is associated with retinal complications: retinoschisis, retinal detachment (RD), which occurs in 6–43% of these patients, serous macular detachment (SMD) and, extremely rarely, choroidal neovascularization (CNV) [5]. Both retinoschisis and RD are linked to the presence of persistent traction of the vitreous on a specific area, the intercalary membrane (ICM). The ICM corresponds to the continuation of the inner retina from the non-colobomatous area to the colobomatous area. Vitreous traction on the ICM and at its edges can lead to schisis defects, micro or mini breaks, allowing fluid to enter the sub retinal space, resulting in RD [14]. RD can impact visual function, need surgical treatment, and has been more stressed in a surgical study group requiring treatment. On the contrary, retinoschisis does not often influence visual function, do not need surgical approach, unless it is complicated by RD.

Pars plana vitrectomy (PPV) is the main treatment to release vitreal traction. In addition, some adjuvant manoeuvres have been described to achieve the goal of retinal reattachment, especially in the case of recurrent RD: laser photocoagulation, applied at the temporal edge of the coloboma, the inner limiting membrane (ILM) graft technique, use of an autologous platelet concentrate, and different tamponade agents such as sulphur hexafluoride gas, standard silicone oil, and heavy silicone oil [15].

In presence of CNV, some case reports describe a good response to anti-vascular endothelial growth factor (VEGF) treatment [16,17]

## 4. Optic Disc Pit

ODP is a rare cavitary congenital anomaly of the optic nerve head, with a prevalence of 1/11,000, unaffected by gender or race [18]. It usually presents as a unilateral greyish oval or round depression, which tends to be located in the temporal segment of the optic disc and can sometimes be associated with ODC [19]; it is bilateral in 15% of patients. It is commonly congenital but can also be acquired and associated with glaucoma and myopia [20]. Like ODC, congenital ODP can also be related to various systemic diseases (Aicardi, Alagille syndrome, neurological developmental malformations) and to specific gene mutations (such as PAX2).

About the pathophysiology, Gass proposed that ODP is an abnormal development of the primordial optic nerve papilla and described it as a mild spectrum of ODC [21]; some histologic reports, confirmed by structural OCT studies, showed a defect in the lamina cribrosa and the presence of dysplastic retinal herniations into the subarachnoid space [14,22]. In addition, Theodossiadis et al. highlighted the anatomical association of ODPs with cilioretinal arteries in up to 64% of cases [23].

At the time of diagnosis, patients often have a normal visual acuity unless they develop macular involvement, with a visual acuity of 20/200 or worse in most untreated patients. As with the other congenital OD anomalies, the diagnosis of ODP is essentially clinical by fundus examination. Imaging techniques can be useful as an aid, particularly to investigate the possible pathophysiology of ODP and its complications.

### 4.1. Multimodal Imaging

In the case of ODP, structural OCT reveals an excavation in the optic nerve head with a hyporeflective area corresponding to the pit. As described by Maertz, OCT in the follow-up of ODPs may show signs of intrapapillary proliferation and a change in pit aspect over time [24]. When macular involvement is present (ODP maculopathy, ODP-M), OCT is useful to assess the presence of fluid, its distribution, and evolution. As Lincoff described, in most cases the fluid follows a specific distribution pattern. First, it causes a schisis-like separation of the inner retina and then reaches the subretinal space, creating a macular neuroepithelial detachment [25]. Currently, OCT helps to determine the distribution and development of retinal fluid and a possible correlation with visual prognosis. Iros et al. described the presence of outer retinal fluid (ORF) in most patients at diagnosis, indicating progression from outer retinal layers to other layers. In 40% of cases, multilayer fluid (MLF) is present, including both outer and intraretinal fluid. He also reported intraretinal fluid in about 78% of patients [26]. Subretinal fluid (SRF) is described as less common than other fluid distribution but correlates with a decrease in vision and deterioration in functional prognosis, especially when associated with subretinal hyperreflective deposits, which are an indicator of chronicity of ODP-M [20,27]. Lamellar macular holes have been reported in association with ODP-M [26].

Fluorescein angiography shows early hypofluorescence at the ODP, followed by late hyperfluorescence when macular involvement is present [28]. Although fluorescein angiography and indocyanine green angiography (if necessary) provide a valuable qualitative image of retinal vascular perfusion, they cannot provide a quantitative assessment of the microvasculature. OCTA can integrate these data. Cennamo et al. reported for the first time the absence of the radial peripapillary microvascular network in both ODP and ODC, resulting in imperfect closure of the embryonic fessure [3]. This report was confirmed by Jiang, who described a decrease in capillary perfusion density within the disc, especially in patients with low visual acuity [29].

### 4.2. Complications

ODP-M may be present in 25–75% of patients between the second and fourth decade of life and consists mainly of a serous macular detachment or macular schisis, which may spontaneously resolve in up to 25% of cases. The origin of the fluid of serous detachment is not clear, but as Michalewski described, it may have a dual origin, both vitreal and cerebrospinal. Among current therapies, pars plana vitrectomy (PPV) is the most recommended method to achieve complete anatomical success, defined as the flattening of the macula without fluid. According to the “European VitreoRetinal society OPTIC PIT STUDY”, SRF responds better to surgical treatment than intraretinal fluid (IRF), with SRF completely or partially regressing in 83% of cases (compared to 73%) 12 months after surgery; in addition, the recurrence rate for SRF is lower than IRF, at 7% and 23%, respectively. Moreover, the same study reported a retreatment rate of 15.6% after 14 months, with SRF completely disappearing in 89% of cases, compared to 67% with IRF. In these cases, the anatomical success and the functional outcomes are not related. After the first surgical time, both anatomical and functional results improve slowly; therefore, special attention must be paid before retreatment. In terms of surgical technique, this study emphasised the fundamental role of PPV with gas tamponade, with endolaser and ILM peeling providing no additional benefit [26]. Two Italian study groups reported results on the use of human amniotic membrane to plug the disc pit during PPV, with improved visual acuity at 12 months and no fluid recurrence [30,31]. Gklava reported the possible use of autologous platelets, both as primary and rescue therapy, with improved functional and morphologic outcomes without relapse over a long follow-up period [32].

A single case of paediatric CNV associated with ODP treated with anti-VEGF injection is reported in the literature [33].

## 5. Morning Glory Syndrome

Morning glory syndrome (MGS) is described as an enlarged optic disc with funnel-shaped excavation, peripapillary pigmentation, radiating distribution of retinal blood vessels and a central white tuft of glial tissue, with a prevalence of 3.6/100,000 children. It is more common in females (2:1 compared to males), is usually unilateral, begins early in childhood, and is associated with extremely poor visual function and prognosis, which is generally less than 0.1 decimal visual acuity [34].

The pathogenesis is unclear: some authors suggest poor development of the posterior sclera and lacrima cribrosa during gestation [35]; recent OCT-based studies have demonstrated the presence of abnormal communication between the subarachnoid and subretinal spaces. In agreement with these data, an abnormality in primary neuroectodermal development with dilatation of the terminal optic stalk has been suggested, followed by a secondary postnatal mesenchymal abnormality [1,36]. Moreover, Cennamo et al. suggest that MGS arises from mesenchymal abnormality due to an increase in the peripapillary network [3]. Extremely rare cases of contractile MGS have been reported. Two main theories have been proposed: the muscle contraction theory, which attributes the contractions to a heterotopic smooth muscle in posterior sclera, and the pressure balance theory, which suggests a fluid flux through a communication between the subarachnoid space and the juxtapapillary subretinal space [37].

Ocular tissues and cerebral vessels are also frequently affected in patients diagnosed with MGS. Persistent fetal vasculature (PVF), cataracts, microphthalmia, retinal detachment, and retrobulbar cysts have been described as ocular complications [38,39,40]. She et al. reported a high prevalence of nonperfused peripheral retina in paediatric MGS and suggested that more attention should be paid to this aspect [41]. About 45% of patients diagnosed with MGS have associated cerebrovascular anomalies that support the hypothesis of a primary mesenchymal defect [42]: basal encephalocele, hypopituitarism, Moya Moya disease, midline cranial defects, and agenesis of the corpus callosum [43]. Moreover, Poillon described a case series of patients with MDS, who also presented an aspect of optic pathway enlargement, which might be a malformation associated with MGS. Therefore, patients found to have MGS should always undergo neuroimaging [44]. Initial clinical manifestation of MGS can have various forms: low visual acuity with posterior segment involvement, leukocoria, microphthalmia, retinal detachment, and, less commonly, strabismus. The diagnosis is clinical and supported by retinal imaging.

### 5.1. Multimodal Imaging

SD-OCT shows increased cup diameter with deep excavation and a raised hyperreflective area corresponding to the presence of glial tissue overlying the optic disc. Cennamo et al. reported OCTA of the peripapillary retina showing a dense microvascular network in the radial peripapillary capillary (RPC), with no vascular difference between the superficial and deep vascular plexus around the optic nerve [45]. Fluorescein angiography shows hypofluorescence in the centre of the disc, numerous radial vessels, and possible peripapillary changes around the optic disc as mottled fluorescence. Ocular echography is a useful tool in patients with PVF associated with MGS and for preoperative planning in cases of RD surgery [46].

### 5.2. Complications

#### 5.2.1. Retinal Detachment

Retinal detachment (RD) is the most common and serious complication in patients with MGS, occurring in approximately one third of patients. It can take an unpredictable clinical course, with spontaneous attachment and re-detachment, and can be rhegmatogenous, tractional, and exudative [47,48]. Currently, the pathogenesis is still unclear: according to Ho et al., an abnormal communication between subretinal and subarachnoid or vitreous compartments seems to be the causative factor. A careful preoperative OCT examination should help the surgeon to detect possible retinal breaks, which have been often described along the margin of excavation [49]. Zou et al. defined some possible risk factors for the development of rhegmatogenous retinal detachment (RRD), such as a deeper excavation with a sharper curve angle at the edge of the excavation, which could result in increased vitreoretinal traction and increased retinal breaks [46]. Considering the poor prognosis after surgery and the need for multiple procedures, only a small number of eyes will undergo surgery, while the remaining eyes should be recommended for regular follow-up. In fact, RD-related MGS is a challenge for surgeons in several aspects, due to a high rate of recurrence, poor visual outcomes, the difficulty to induce posterior vitreous detachment and to perform a complete vitreous removal, the possibility to perform peeling of the epiretinal membrane (ERM) and the ILM, the iatrogenic induction of retinal tears during fibroglial tissue removal, the use of perfluorocarbon fluids (PFCLs) that can migrate into both the subretinal and the subarachnoid space, and the high rate of proliferative vitreoretinopathy (PVR) [50,51,52]. Some adjuvant treatments can be performed during pars plana vitrectomy, but there are no clear results in the literature. For example, laser photocoagulation at the optic disc margin in the absence of obvious retinal breaks is controversial because it could damage the nerve fibres at the papillomacular bundle; therefore, diode laser may be preferable to preserve the nerve fibre layer. Some procedures have been described as possible treatments for recurrent RD to close peripapillary holes: packaging with ILM or the use of a human amniotic patch placed directly on the optic nerve [53,54]. There have been some concerns about the potential communication between the subretinal space or vitreous cavity and the subarachnoid space when using silicone oil as a tamponade because of the risk of migration of emulsified bubbles [55]. Preoperative surgical planning with OCT, especially the three-dimensional remodelling scan, is fundamental for the correct selection of patients to undergo surgery and to achieve a better outcome. Some reports have described spontaneous resolution of RD in patients with only serous components, without retinal breaks [56]. Prakash et al. described the use of oral acetazolamide as a possible treatment for serous macular detachments [57].

#### 5.2.2. Choroidal Neovascularization

A rare complication of MGS is the development of CNV, both in adults and children. It typically occurs at the edge of the staphyloma and is associated with sudden painless visual decrease. The diagnosis is made clinically and supported by imaging examinations: OCT, fluorescein, and indocyanine green angiography [58]. Cennamo et al. emphasised the usefulness of OCT angiography for CNV-related MGS diagnosis, especially in patients with possible misdiagnosis [59]. Although there is no established regimen, anti-VEGF therapy is indicated for treatment [60].

## 6. Conclusions

This comprehensive review highlights the current state of knowledge of congenital cavitary optic nerve anomalies in terms of pathophysiology, clinical assessment, potential treatment, and associated complications.

Despite being a spectrum of rare disorders, these congenital anomalies are associated with a significant impact on the medical history of the patient, who is diagnosed with them. Indeed, in ophthalmology, these conditions lead to significant visual impairment, long follow-up periods (from childhood to adulthood), and the need for repeated medical and, especially, surgical treatments. In addition, the diagnosis of MGS or ODC, compared to the associated systemic diseases, requires the involvement of the patient in a multidisciplinary approach, with several medical experts and complementary examinations, such as neurological imaging.

Congenital anomalies are a challenge both in research and in clinical practice. At present, we still have imprecise data on the pathophysiology and researchers are trying to elucidate the pathological mechanisms using animal models. Several gene mutations have been described, but the pathways of optic nerve head abnormalities are still not clear. Regarding clinical aspect, non-invasive exams such as OCT and OCTA have been used to study morphological aspects of the optic nerve, characterize these abnormalities, and try to define the basis for the development of the associated complications. These data may be fundamental for the development of an improved treatment approach, i.e., for the selection of patients to be treated in case of complications and for the definition of the best treatment approach, especially in cases of RD.

In conclusion, congenital anomalies of the optic nerve represent a major challenge for ophthalmologists and medical professionals. Further research and collaboration between scientists and clinicians can clarify the pathophysiology of these diseases and define a correct and specific treatment approach.

## Data Availability

Not applicable.
